# Clinicopathological and prognostic significance of COX-2 in glioma patients: a meta-analysis

**DOI:** 10.1055/s-0042-1758864

**Published:** 2022-12-29

**Authors:** Jun Wang, Chenyan Xiang, Yi Cai, Ziyi Mei, Qianqian Lu, Binbin Liu, Lili Zou

**Affiliations:** 1 China Three Gorges University, The People's Hospital, Yichang, Hubei Province, China.; 2 China Three Gorges University, College of Basic Medical Sciences, Hubei Key Laboratory of Tumor Microenvironment and Immunotherapy, Yichang, Hubei, China.; 3 China Three Gorges University, College of Basic Medical Sciences, The Institute of Infection and Inflammation, Yichang, China.

**Keywords:** Cyclooxygenase 2, Glioma, Meta-Analysis, Survival, Ciclo-oxigenase 2, Glioma, Metanálise, Sobrevida

## Abstract

**Background** In recent years, cyclooxygenase-2 (COX-2) has been identified as a cancer stem cell (CSC) marker in gliomas. Nevertheless, the clinical and prognostic significance of COX-2 in glioma patients remains controversial.

**Objective** To evaluate the correlation of COX-2 with the prognosis in glioma patients.

**Methods** Eligible studies on this subject were included, and pooled odd ratios (ORs) and hazard ratios (HRs) with 95% confidence intervals (95%CIs) were estimated. Publication bias was assessed through funnel plots, and heterogeneity and sensitivity were analyzed as well.

**Results** In the present study, 11 articles with a total of 641 patients were included. The high expression of COX-2 in glioma patients was negatively associated with overall survival (OS) (n = 11; HR = 2.26; 95%CI = 1.79–2.86), and the subgroup analysis showed no differences in OS between Asian (n = 5; HR = 2.16; 95%CI = 1.57–2.97) and non-Asian (n = 6; HR = 2.39; 95%CI = 1.69–3.38) glioma patients. The Begg funnel plots test indicated that there was no evident risk of publication bias in the meta-analysis.

**Conclusion** The present study suggests that COX-2 could be recommended as a useful pathological and prognostic biomarker in the clinical practice.

## Resumo

**Introdução** Nos últimos anos, a ciclooxigenase-2 (COX-2) foi identificada como um marcador de células-tronco cancerígenas (CSC) em gliomas. No entanto, o significado clínico e prognóstico da COX-2 em pacientes com glioma permanece controverso.

**Objetivo** Avaliar a correlação da COX-2 com o prognóstico em pacientes com glioma.

**Métodos** Estudos elegíveis sobre este assunto foram incluídos e foram estimados *odds ratios* (ORs) e *hazard ratios* (HRs) com intervalos de confiança de 95% (IC 95%). O viés de publicação foi avaliado por meio de gráficos de funil, e a heterogeneidade e a sensibilidade também foram analisadas.

**Resultados** No presente estudo foram incluídos 11 artigos com um total de 641 pacientes. A alta expressão de COX-2 em pacientes com glioma foi negativamente associada à sobrevida global (OS) (n = 11; HR = 2,26; IC 95% = 1,79-2,86), e a análise de subgrupo não mostrou diferenças na OS entre asiáticos (n = 5; HR = 2,16; IC 95% = 1,57–2,97) e não asiáticos (n = 6; HR = 2,39; IC 95% = 1,69–3,38) pacientes com glioma. O teste de gráficos de funil de Begg indicou que não havia risco evidente de viés de publicação na metanálise.

**Conclusão** O presente estudo sugere que a COX-2 pode ser recomendada como um biomarcador patológico e prognóstico útil na prática clínica.

## INTRODUCTION

Gliomas are the most common primary human intracranial tumors, and their annual incidence is of approximately 6 cases per every 100 thousand individuals worldwide. 1 Diffuse gliomas occur in both adults and children and make up about 30% of all brain and central nervous system (CNS) tumors, and 81% of all malignant brain tumors. 2 In children, low-grade gliomas (LGGs) predominate, whereas high-grade diffuse infiltrating gliomas (DIGs) are more common in adults. The World Health Organization (WHO) incorporates molecular parameters in addition to histology to define five primary designations of adult diffuse glioma: glioblastoma, isocitrate dehydrogenase (IDH) wild type; glioblastoma, IDH mutant; diffuse or anaplastic astrocytomas, IDH wild type; diffuse or anaplastic astrocytomas, IDH mutant; and oligodendroglioma or anaplastic oligodendroglioma, IDH mutant, and 1p19q co-deletion. 3 4 Despite various treatments for glioma, such as surgery, chemotherapy, or radiotherapy, the 5-year survival rate is lower than 10%, 5 6 and the median overall survival (OS) is only of about 12 to 14 months. 7 Therefore, there is an urgent need to seek effective therapeutic targets and develop new therapeutic strategies.

Cyclooxygenase (COX) is the rate-limiting membrane bound enzyme of prostaglandins (PGs), which have three isoforms, designated COX-1, COX-2, and COX-3. 8 The COX-2 isoform is induced by various stimuli such as inflammatory signals, mitogens, cytokines, and growth factors that are constitutively expressed at low to moderate levels in cells. 9 10 11 12 Alhouayek and Muccioli 13 found that COX-2 could result in the generation of PGs from arachidonic acid (AA) in neoplastic and inflamed tissues. In the brain, COX-2 is located in neurons of the neocortex and hippocampus, and it could be induced by cell cytokines, growth factors, and tumor promoters. 14 15 Since it is associated with carcinogenesis, oncogenesis, and tumor progression, COX-2 can act as a prognostic predictor in different human cancer types. Once it has been inhibited, the growth of the tumor will be attenuated. The expression of tumor-cell proliferation markers will be decreased, resulting in apoptosis. 16 17 Numerous investigators have evaluated the expression of COX-2 in various cancers, including prostate cancer, 18 breast cancer, 19 hepatocellular cancer, 20 non-small cell lung cancer, 21 gastrointestinal malignancies, 22 hematological cancer, 23 and head and neck tumorigenesis. 9 However, there is still insufficient clinical data to determine the clinical significance of COX-2 in gliomas. Research on that may help us identify new targets that enable us to develop effective prognostic predictors and a therapeutic approach to this challenging solid malignancy. A systematic analysis of the published studies was performed to elucidate whether COX-2 expression correlates with the prognosis in glioma patients.

## METHODS

### Reference search

A search for studies published from June 1994 to June 2021 was conducted with no restrictions (in terms of language, origin, time, period, and population) in the following electronic literature databases: PubMed, EMBASE, MEDLINE, Web of Science, Google Scholar, CNKI, and WanFang. The search terms were used in the the following combinations: ( *cyclooxygenase-2* or *COX-2* or *prostaglandin-endoperoxide synthase 2* or *PTGS-2* or *PHS-2* or *PGG/HS* or *hCox-2* or *GRIPGHS* ) and ( *gliomas* or *glioma* or *glioblastoma* or *glioblastoma* *multiforme* or *GBM* or *diffuse infiltrating gliomas* or *DIG* or *astrocytomas* or *astrocytoma* or *ependymocytoma* or *medulloblastoma* or *oligoastrocytoma* or *oligodendroglioma* or *anaplastic-astrocytoma* ) and ( *prognosis* or *prognostic* or *expression* or *regulation* or *upregulation* or *downregulation* or *outcome* or *survive* or *survival* ). Furthermore, potential eligible publications were screened from the relevant reference lists, and supplemental data were further investigated manually if essential data was unavailable from the original references.

### Eligibility criteria

All potential eligible publications in this meta-analysis were assessed by two individuals (JW and YC) separately, and any divergences were resolved by further discussion. The studies included had to meet the following criteria: 1) patients with a clinical diagnosis of glioma regardless of the subtype; 2) case-control experiment design; 3) the main outcomes of interest were OS, progression-free survival (PFS), and WHO grade, also considering age and gender; 4) focus on exploring the relationship between COX-2 and glioma that provided sufficient information on the OS, PFS, and clinicopathological indicators; 5) COX-2 expression data can be used to calculate the hazard ratio (HR) or relative risks (RRs) with 95% confidence intervals (95%CIs); and 6) the expression level of COX-2 was evaluated by immunohistochemistry (IHC), tissue microarray (TMA), real-time polymerase chain reaction (RT-PCR), or Western blot (WB). Studies meeting the following criteria were excluded: 1) abstracts only, comment articles, letters, editorials reviews; 2) studies not conducted with human subjects; 3) studies reanalyzing published data; 4) insufficient data; and 5) in studies with the same population, the one with smallest sample.

### Data extraction and quality assessment

To reduce the bias and enhance the credibility, data extraction was conducted by two independent reviewers (JW and YC) using the following standardized criteria: name of the first author, country of origin of the study population, year of publication, sample size, histological type, study methods, WHO grade, adjusted factors, and data to calculate HRs with 95% CIs. The quality of the included studies was assessed using the criteria of the Newcastle–Ottawa Scale (NOS). 24 According to the NOS scale, there were 4 points for the selection of patients, 2 points for comparability, and 3 points for exposure-factor measurement. Studies that scored a total of 6 or more points were defined as high-quality, while those that scored 5 or fewer points were defined as low-quality.

### Statistical analysis

The meta-analysis was performed using the R package meta (R Foundation for Statistical Computing, Vienna, Austria), version 4.18. 25 The WebPlotDigitizer (version 4.4, Ankit Rohatgi, Pacifica, California, United States) was used to extract data from tables, text, and/or figures. In addition, methods described by Tierney et al. 26 and Parmar et al. 27 were used in this analysis.

To assess the heterogeneity among the different studies, the Chi-squared test and the Q test 28 were used. If the heterogeneity was significant (defined as *p* > 0.05), a random-effects model would be performed; meanwhile, if no statistical heterogeneity was found, a fixed-effects model could be used. Subgroup analyses were mainly performed based on Asian and non-Asian regions. For the subgroup analysis of studies with adjusted estimates, we would assess whether Asian and non-Asian ethnicities have an impact on the survival of glioma patients. The Begg funnel plots test was used to assess the publication bias, 29 and sensitivity analysis was applied to estimate the influence of a single study on the overall evaluation.

## RESULTS

### Study selection and description of included studies

**Figure 1:**
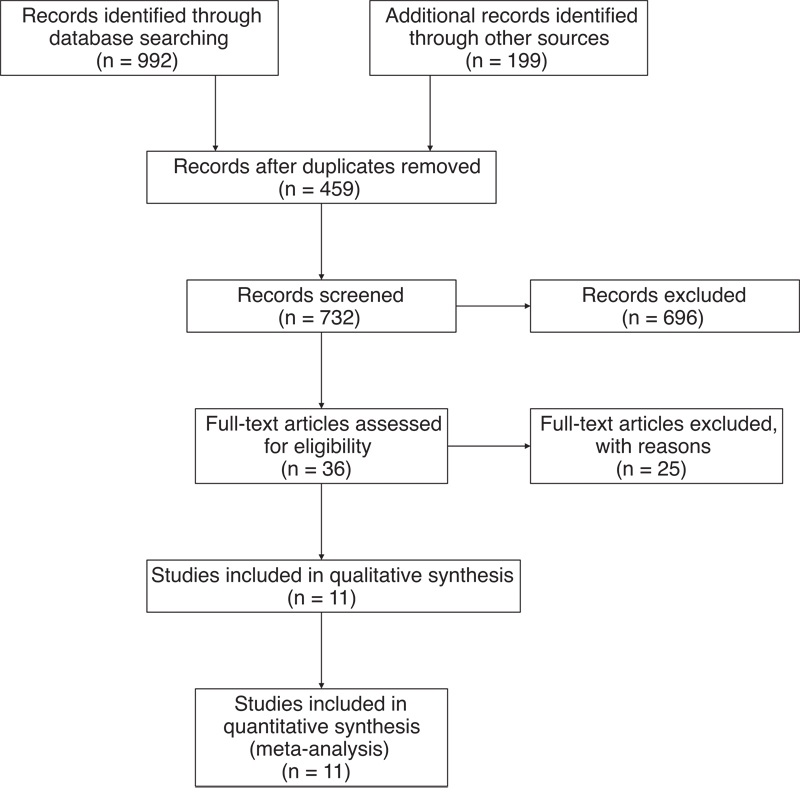
Flow chart of the literature search and selection of articles.

The search steps are described in detailed in Figure 1 . First , 1,191 papers were selected based on the aforementioned inclusion criteria, and 459 articles were excluded because they were duplicates, as well as 696 articles that were not relevant to the study based on the titles and abstracts. The remaining 36 articles were further assessed by 2 observers, who excluded the following types of study: reviews, commentaries, conference presentations, articles not related to COX-2, and those that did not provide sufficient data. Eventually, 11 eligible articles were included.

All 11 studies, which were published from 2001 to 2017 and involved a total sample of 641 patients, are listed in Table 1 . Six studies were conducted in non-Asian populations and five, in Asian populations, including two from Korea and three from China. Eight studies included reported the OS, one reported the median survival (MS), one reported the HR, one reported a summary of the clinical data, and 1one indicated the PFS. Patients with positive COX-2 were evaluated by IHC (10 studies) and TMA (1 study). All detected specimens were derived from glioma tissues via surgical resection. Four studies reported adjusted estimates with different confounding factors, and most studies 37 38 39 41 42 44 45 46 47 with adjusted estimates were high-quality.

**Table 1:** Characteristics in 11 included studies

Study ID	Country	Patients	Histology	Grade	Method	Outcomes	Confounding factors	Quality
Shono et al. (2001) 37	United States	66	Glioma	Not available	Immunohistochemistry	Overall survival	Age, histology, MIB-1, p53, Rb, and BCL2	7
Castilla et al. (2003) 38	United Sates	60	Glioma	II-III	Immunohistochemistry	Overall survival	None	6
Lee et al. (2004) 39	Korea	25	Glioma	Not available	Immunohistochemistry	Overall survival	None	6
Buccoliero et al. (2004) 40	Italy	34	Glioma	II-IV	Immunohistochemistry	Median survival	None	4
Perdiki et al. (2007) 41	Greece	83	Astrocytomas	II-IV	Immunohistochemistry	Overall survival/hazard ratio	Age, WHO grade, gender, VEGF, HIF-1α, MVD, TVA	7
Onguru et al. (2008) 42	Turkey	54	Glioblastoma	IV	Immunohistochemistry	Hazard ratio	None	6
EI-Sayed and Taha (2011) 43	Egypt	26	Glioblastoma	II-IV	Immunohistochemistry	Summary of clinical data	None	5
Myung et al. (2010) 44	Korea	56	Glioma	I-IV	Tissue microarray	Overall survival/progression-free survival	WHO grade, Snail, treatment, extent of surgery	8
Chen et al. (2012) 45	China	91	Glioma	Not available	Immunohistochemistry	Overall survival	None	6
Wang et al. (2015) 46	China	76	Glioblastoma	Not available	Immunohistochemistry	Overall survival	Age, gender, 5-LO, Ki-67, p53, EI	8
Zhang et al. (2017) 47	China	70	Glioma	I-IV	Immunohistochemistry	Overall survival	None	6

Abbreviations: MIB-1, Mindbomb 1 E3 ubiquitin ligase; p53, tumor protein p53; Rb, retinoblastoma protein; BCL2, B-cell lymphoma 2, an apoptosis regulator; VEGF, vascular endothelial growth factor; HIF-1alpha, hypoxia inducible factor 1 subunit alpha; MVD, microvessel density; TVA, total vascular area; 5-LO, 5-lipoxygenase; Ki-67, marker of proliferation Ki-67; EI, edema index.

### Impact of COX-2 on the overall survival of glioma patients

**Figure 2:**
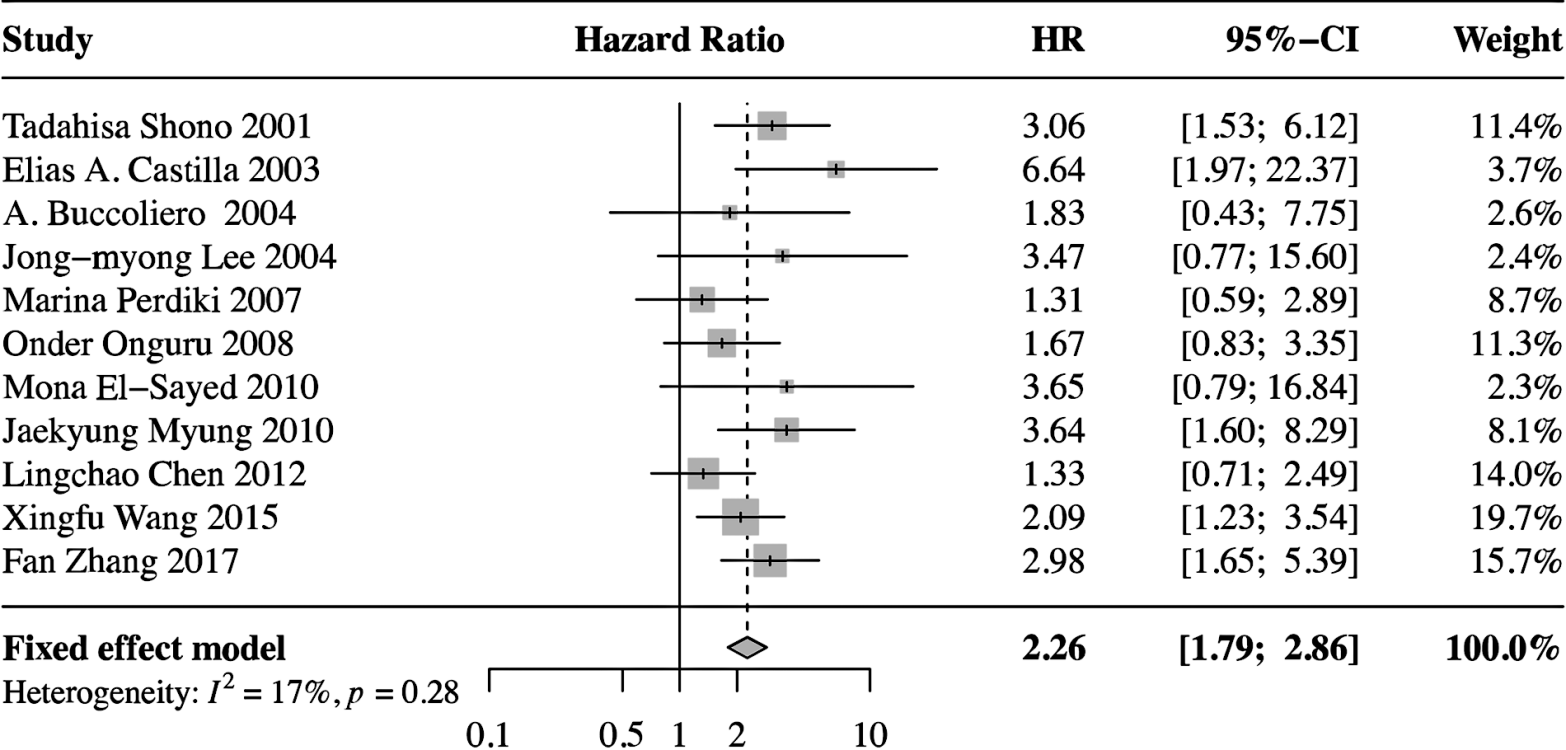
Fixed-effects model showing an association between high COX-2 expression and poorer OS in glioma patients. High COX-2 expression was negatively associated with OS (n = 11; HR = 2.26; 95%CI = 1.79–2.86).

**Figure 3:**
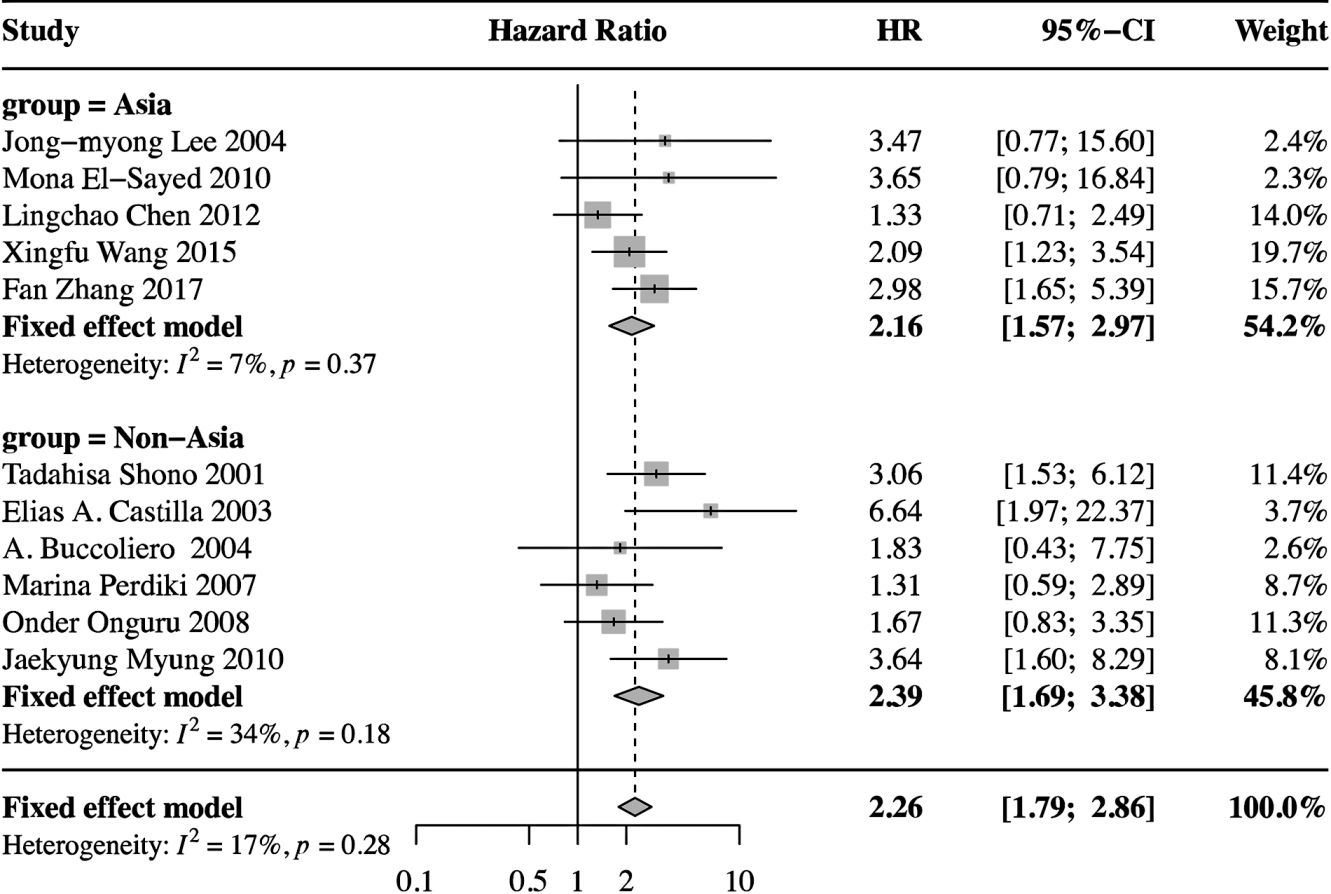
Subgroup analysis showing no differences in OS between Asian (n = 5; HR = 2.16; 95%CI = 1.57–2.97) and non-Asian (n = 6; HR = 2.39; 95%CI = 1.69–3.38) glioma patients.

To further evaluate the relationship between COX-2 and prognosis in postoperative glioma patients, a survival analysis of the OS was conducted. The heterogeneity among the included studies was low ( *I* ^2^ < 50%; *p* -value of *Q* test  > 0.1), so the fixed-effects model was used to calculate the pooled HR. Figure 2 shows that high COX-2 expression was negatively associated with OS (n = 11; HR = 2.26; 95%CI = 1.79–2.86). The subgroup analysis showed no differences in terms of OS between Asian (n = 5; HR = 2.16; 95%CI = 1.57–2.97) and non-Asian (n = 6; HR = 2.39; 95%CI = 1.69–3.38) glioma patients ( Figure 3 ).

### Publication bias and sensitivity analysis

**Figure 4:**
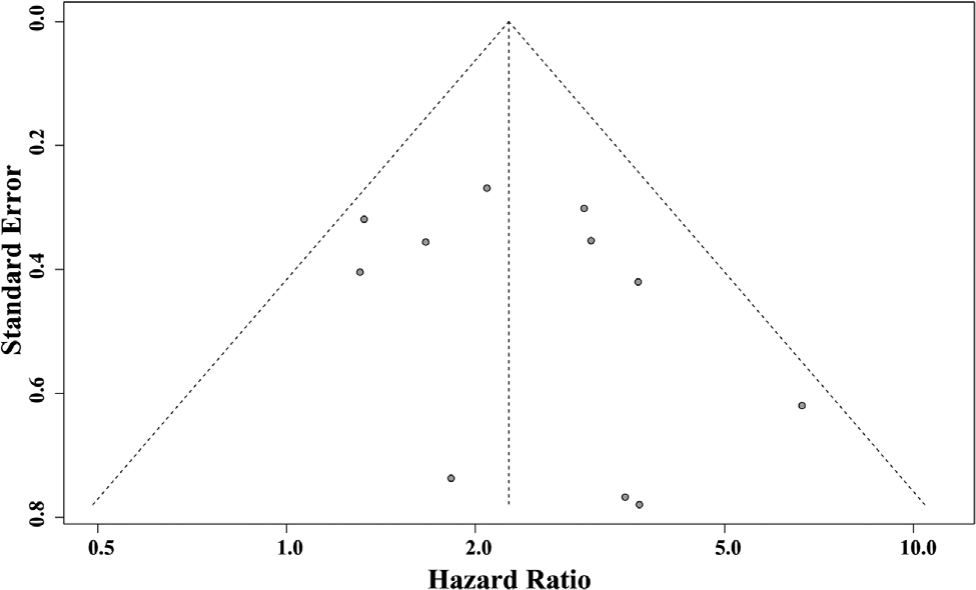
Funnel plot for the meta-analysis of the association between COX-2 expression and OS in glioma patients. The data points formed a roughly symmetrical, upside-down funnel and, the *p* -value of the Egger test was of 0.2826, which indicates no publication bias.

**Figure 5:**
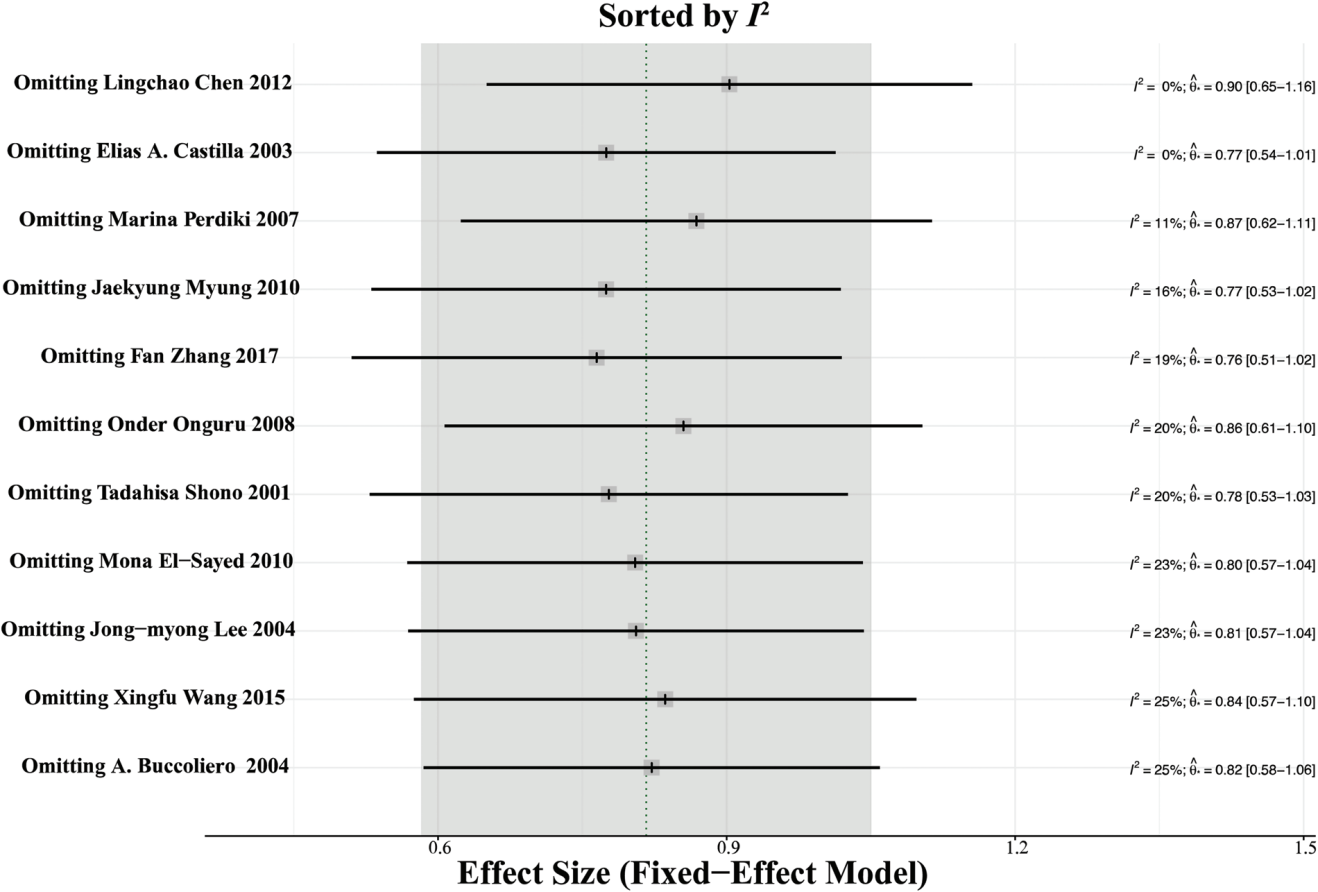
Sensitivity analysis sorted by the *I* ^2^ value of the leave-one-out meta-analysis indicating the lack of influential cases.

The small-study effects were inspected through a funnel plot, and the Egger regression test was used to test the asymmetry of the funnel plot asymmetry. As shown in Figure 4 , the data points formed a roughly symmetrical, upside-down funnel and, the *p* -value of the Egger test was of 0.2826, which indicates the lack of publication bias in the present meta-analysis. Leave-one-out sensitivity meta-analysis was performed to detect the studies which most influenced the overall estimate of our meta-analysis ( Figure 5 ), and the result indicated no influential cases.

## DISCUSSION

Gliomas are the most common tumors of the CNS. The most conclusive prognostic factors for glioblastoma are the extent of the tumor resection, age at diagnosis, and the Karnofsky performance status. The importance of PGs and COX-2 in the formation and progression of gliomas has been suggested by early studies and correlations between increased PG synthesis and tumor grade have been observed as well. 30 31 Although recent studies have reported significant relationships between shortened patient survival and elevated COX-2 expression in various cancers, 32 33 34 35 to our knowledge, such a relationship between COX-2 expression and patient survival in gliomas has not been determined. In the present study, we have performed a systematic meta-analysis to evaluate the association between COX-2 and OS in patients with glioma.

Eleven eligible studies were identified and included, and the pooled HRs and RRs with 95%CIs were calculated. The outcomes of 641 patients with glioma, as well as the prognosis, and pathology were summarized. The OS analysis showed an obvious correlation between high COX-2 expression and poor 5-year OS (n = 11; HR = 2.26; 95%CI = 1.79–2.86). Thus, COX-2 may be considered a novel pathological and prognostic biomarker for clinical use.

Several limitations of the present analysis should be noticed. In 10 out of the 11 included studies, COX-2 expression was detected by IHC. Only 1 study analyzed it using TMA. Though a traditional method, the outcomes of IHC may be affected by different primary antibody clones and concentrations. However, we cannot perform a subanalysis by different antibodies to evaluate the underlying bias of the method on the pooled ORs. In addition, the definition of the cut-off value among the studies also varied, which can also lead to bias. Thus, every factor that may have affected the analysis should be fully considered.

As publication bias is the major cause of bias in systematic meta-analyses, it should be calculated. In the present study, publication bias was assessed by the Begg funnel plot test, 36 and no evident risk was found. Other factors may also lead to bias, including language. The articles included in the present study were written in English and Chinese; therefore other potentially eligible studies were not included, which may also have led to bias.

Based on the results of the present study, COX-2 is a potential clinical biomarker in glioma patients with poor prognosis. The results indicated that COX-2 has significance in the pathological diagnosis and prognostic prediction of glioma patients in the clinical practice. In addition, given the limitations of the current analysis, well-designed prospective clinical studies are required to further evaluate the role of COX-2 in the selection of a therapeutic approach in glioma.
